# Rhythms, Patterns and Styles in the Jaw Movement Activity of Beef Cattle on Rangeland as Revealed by Acoustic Monitoring

**DOI:** 10.3390/s25041210

**Published:** 2025-02-17

**Authors:** Eugene David Ungar, Ynon Nevo

**Affiliations:** 1 Department of Natural Resources, Institute of Plant Sciences, Agricultural Research Organization (ARO), Volcani Center, 68 HaMaccabim Road, P.O. Box 15159, Rishon LeZion 7505101, Israel; 2 Department of Animal Science, The Robert H. Smith Faculty of Agriculture, Food & Environment, The Hebrew University of Jerusalem, Herzl 229, Rehovot 7610001, Israel; ynonevo@gmail.com

**Keywords:** bite, chew, grazing behavior, ingestion, Mediterranean zone, rumination, signal processing

## Abstract

Grazing shapes rangelands globally, but it is difficult to study. Acoustic monitoring enables grazing to be described in terms of jaw movements, which are fundamental to how herbivores interact with their foraging environment. In an observational study on Mediterranean herbaceous rangeland, 10 beef cattle cows were monitored continuously over multiple days in two seasons. The algorithm used to analyze the acoustic signal furnished (without classification) a data sample of ≈5 M ingestive and ruminatory jaw movements. These were analyzed as between-event intervals and as minutely rates. The rumination displayed a consistent, strong rhythm and pattern of jaw movements. In contrast, there was no single “signature” jaw movement pattern for grazing (i.e., non-rumination). Although the underlying natural rhythm of rumination dominated non-rumination, it was intermittently and irregularly interrupted by longer intervals, whose size scaled logarithmically. There was evidence of further substructure, with a degree of separation between “grazing” and “resting” in the conventional sense. Three broad grazing styles emerged. In the “intense” style, animals sustained long runs of jaw movements in the natural rhythm, with relatively few interruptions. In the “regular” style, comprising the majority of non-rumination jaw activity, the natural rhythm still dominated, but was punctuated at irregular intervals by eruptions of somewhat longer intervals. The “diffuse” style comprised shorter runs in the natural rhythm, punctuated by highly erratic intervals spanning orders of magnitude. When the jaw movement events were viewed as minutely rates, the non-rumination population showed strong bimodality in the distribution of non-zero rates, with peaks at ≈60 and ≈15 jaw movements min^−1^, suggesting two modes of grazing. The results strongly support the notion of behavioral grazing intensity and call into question the approach of viewing grazing as a binary state or expecting measures of grazing time to be strongly indicative of intake rate. Rate- and interval-based analyses of information at the jaw movement level can yield a penetrating profile of how an animal interacts with its foraging environment, epitomized in a graphical formulation termed the time accumulation curve. These results strengthen the case for the further development of this sensor technology.

## 1. Introduction

The foundations of sustainable rangeland management practices lie in our understanding of the key rate processes of the system: plant growth and animal consumption. On the plant side, technologies such as remote sensing have transformed our ability to study rangeland vegetation [1,2]. On the animal side, in contrast, the study of grazing by herbivores on rangeland has lagged in terms of sensor technology, which in turn hinders progress in how we describe and conceptualize the process itself [3]. Intake, in particular, remains challenging to study and quantify, despite its centrality to vegetation dynamics, animal nutrition and overall system stability [4,5].

One way of “eavesdropping” on intake is via the biting and chewing sounds it generates, as demonstrated in cattle decades ago (e.g., [6]). The potential utility of acoustic monitoring becomes apparent from considering the basic properties of the intake process. Herbage intake is the outcome of a set of behaviors, of which a central one is movement (opening and closing) of the jaws to perform bite and chew actions, both of which generate sound. The potential scope of how an animal performs jaw movements is determined by anatomy and biomechanics [7,8,9,10], but its expression is profoundly influenced by forage conditions, including the abundance, spatial distribution and quality of the herbage [11,12]. Assuming for the moment that grazing is a binary state (the animal is either grazing or not grazing), a simple arithmetic definition of daily herbage intake rate is the product of daily active grazing time and intake rate during active grazing. Intake rate during active grazing can be expanded to the product of bite rate during active grazing and bite weight. Bite rate during active grazing can, in turn, be expanded to the product of jaw movement rate during active grazing and the proportion of jaw movements allocated to biting actions [13].

While it is true that the measurement of bite mass is relatively intractable, the remaining terms in the above expression are tractable empirically, although not equally so. In light of the centrality of jaw movements, various sensors have been proposed to monitor them, as reviewed by Andriamandroso et al. [14]. In acoustic monitoring, but probably more generally too, the detection of jaw movements is easier than their classification, especially when the chew–bite type of jaw movement is prevalent, as expected in cattle grazing [15]. Crucially, by defining grazing in terms of the fundamental behavioral component of the process—the jaw movement—the functional connection that must exist between it and the mass component of intake, via chewing, can be leveraged [16,17]. Even without knowing the coefficient by which to convert chewing actions into units of mass, it is reasonable to argue that, under a given set of forage conditions, the short-term jaw movement rate is strongly coupled to the short-term bite rate, which is in turn coupled, perhaps less strongly, to short-term intake rate [18,19].

As a quantitative baseline for comparison, itself based on acoustic monitoring, consider the single-group study of Ungar and Rutter [20], in which six dairy cows grazed under ideal conditions that placed no constraints on herbage abundance or quality. During a 10 min sampling interval of active grazing, the cows performed, on average, 69 jaw movements min^−1^. Although there were sporadic pauses in the rhythm of the jaw activity, the point is that these animals grazed at a jaw movement rate that was close to its potential, unimpeded “cruising speed”, the entire (albeit limited) time.

Free-ranging livestock on extensive rangelands are probably near the opposite extreme of forage conditions in terms of the variability expected to be elicited in short-term grazing behavior, for two reasons. First, uninterrupted access to the forage resource may be associated with more grazing time at a lower behavioral intensity of grazing (presupposing that “grazing intensity” in this sense exists). Second, and perhaps more importantly, forage conditions on extensive rangelands are poorer than those encountered by dairy cows on pasture on every score: overall abundance may be an order of magnitude lower; nutritional quality would mostly be lower; and the spatial heterogeneity of abundance and quality might be an order of magnitude greater. Consequently, one might expect a more diffuse distribution of short-term jaw movement rate, as would be revealed by examining the timeline of jaw activity as rates.

A second, and more mechanistic, way of defining intake is functionally. The simplest functional definition of intake rate is based on the time budget of the process: the mass harvested in a bite in relation to the search and handling times invested [21]. For the dairy cows above, performing 69 jaw movements min^−1^, search time was effectively zero because sward uniformity was high and selectivity was close to zero. A high number of jaw movements performed per minute is indicative of unconstrained forage conditions, but the decisive factor is rhythm, i.e., the regularity of successive jaw movement events, as demonstrated by the time interval between them [22,23,24]. Based on a companion, but smaller, acoustic monitoring study, conducted with goats under extensive forage conditions [25], it was proposed that as the search time component of the intake process grows, the interval between successive jaw movements will be punctuated with larger values. Thus, a reduction in jaw movement rate below the unconstrained maximum is not the result of a general slowing down while maintaining a regular rhythm, but the fracturing of the unconstrained rhythm with interruptions. This may give rise to higher-level patterns, as would be revealed by interval-based analysis of the timeline of jaw activity.

The above background indicates that a description of grazing at the jaw movement level, as afforded by acoustic monitoring, should facilitate a deeper understanding of how herbivores interact with their foraging environment. We sought to advance this approach by monitoring the jaw activity of free-ranging beef cattle on Mediterranean herbaceous rangeland over multiple days. The detection of jaw movements acoustically is still in its infancy, and a commercially available sensor has yet to be developed. There were adequate resources to monitor all individuals in a single-group, observational study with no treatment structure. Obviously, jaw movement events are not independent and are highly serially correlated. Likewise, animals in a paddock are not independent in their behavior. For such reasons, the database is, strictly speaking statistically, a single data point or sample. But it is a well-grounded one, comprising ≈5 M jaw movement events from multiple animals, sampled continuously over multiple days, in two contrasting seasons. The rate-based and interval-based approaches introduced above were applied to this collection of events to profile how free-ranging animals interact with their foraging environment.

## 2. Materials and Methods

### 2.1. Study Site

The study was conducted on an area of Mediterranean herbaceous rangeland in the region south of Mt. Carmel, Israel, inside the UNESCO-recognized Biosphere Reserve of Megiddo (https://www.unesco.org/en/mab/megiddo?hub=66369, accessed on 5 January 2025), and under the stewardship of kibbutz Ein HaShofet. The topography comprises rolling hills with a mean altitude of 300 m above sea level. The soils are predominantly shallow and of the rendzina type that developed over a soft chalky bedrock [26]. The climate is strongly seasonal with hot, dry summers and cool, rainy winters, with mean annual rainfall of 600 mm (for a hydrological cycle starting 1 October) and average daily temperature of 18 °C. The species-rich vegetation of the rangeland is primarily herbaceous and dominated by annuals, interspersed with patches of low shrubs [27]. The paddocks are bordered by planted windbreaks. The annual grazing cycle commences in December/January, approximately one month after the emergence of the annual herbaceous vegetation, a process triggered by the first major rains of the hydrological cycle. The rangeland paddock used in this study was of a prevailing southerly aspect with moderate slopes of 3–7%.

### 2.2. Animals and Their Management

The experimental animals were drawn from a mixed-breed, beef-cattle herd of 800 head. The primary calving season was in the summer (June–August), and calf weaning was at age 6–8 months. To minimize disturbance to the herd as a whole, 12 medium-sized mature cows (≈520 kg live weight), which did not display excessive agitation in the animal handling facilities, were separated and moved to the experimental paddock on 19 October 2012. The fenced paddock was equipped with troughs for water and supplementary feeding, as well as animal handling facilities. A bull was introduced two days later, at the start of the breeding season. The 12 cows had a number of months in which to form a stable social group and familiarize themselves with the paddock before the acoustic monitoring began. Poultry litter was provided *ad libitum* in the summer months as a source of non-protein nitrogen.

### 2.3. Acoustic Monitoring Periods and Environmental Conditions

The acoustic monitoring of the 12 experimental animals was conducted in the spring and in the summer of 2013, when the cows were in mid- and high pregnancy, respectively. Monitoring in the spring commenced on 21 March, at the height of the season in terms of herbage growth and quality. The summer, dry season monitoring commenced on 4 July, when the quality of the herbage was low, but before grazing had depleted it substantially (see photographs in Supplementary Figure S1). Meteorological data for the relevant monitoring periods in the two seasons were obtained from a public database (Israel Meteorological Service, Beit Dagan, Israel; https://ims.gov.il/en/data_gov, accessed on 5 January 2025). There was no rainfall during either monitoring period. The mean daily temperature in the spring and summer monitoring periods was 15.3 °C and 24.5 °C, respectively. Daily rainfall for the 2013/2014 hydrological year, and the 10-minutely timeline of environmental conditions during the two monitoring periods are shown in Supplementary Figures S2–S7. The average sunrise time in the spring and summer monitoring periods was 05:40 h and 04:43 h, respectively, and the corresponding sunset times were 17:54 h and 18:47 h (times throughout are UTC+2).

### 2.4. Sensor Design Considerations

Methodologically, obtaining acoustic data from free-ranging beef cattle is much more challenging than doing so in short-duration trials conducted with relatively docile dairy cows (as in [20]). Beef cattle need to be corralled and then coaxed through a cattle chute and into a cattle squeeze for safe handling. Aside from being time-consuming and disturbing to the daily behavioral rhythm of a free-ranging animal, all the steps of this process are stressful for the animal. That necessitates incorporating into the sensor design sufficient power to sustain continuous monitoring for multiple days. Furthermore, makeshift arrangements for microphone placement on the head of the animal, such as on the forehead as used by Ungar and Rutter [20], would not suffice under free-ranging conditions. A halter-based sensor design is feasible, but has disadvantages; not least, the microphone would be on the nasal bridge, making it susceptible to physical damage. The approach chosen for the present study was to attach the sensor to one horn of the animal. This microphone location was found by Tani et al. [28] to yield a high-quality signal, which we confirmed in a preliminary field test of the sensor that was assembled here.

### 2.5. Acoustic Sensor Components

The acoustic sensor was built around a commercially available MP3 device (Sansa Clip+, SanDisk, Milpitas, CA, USA), which had a record mode and a slot for a MicroSD memory card. The device was small and of rectangular shape (54.5 × 34.5 × 10.4 mm; rear clip removed), lightweight (24 g) and found to be robust under field conditions. A number of modifications were made to the device in house. The built-in microphone was detached and discarded, and two wires, of 4 cm length, were soldered to the motherboard connectors in its place. To the other end of the wires was attached a 2-pin Molex-type female connector. In order to connect to an external voltage source, an additional pair of wires, of 4 cm length, were soldered to the motherboard connectors to which the internal battery was, and remained, connected. A 2-pin Molex-type male connector was attached to the other end of the wires. An aperture was cut into the device casing to accommodate both pairs of wires and enable the casing to be fully shut. An external vibration-type piezoelectric microphone (Model wcp500, Cherub Technology Co., Nanshan, China) provided the source signal to be recorded. The original cable was shortened to 10 cm and terminated by a 2-pin Molex-type male connector. The external voltage source was a rechargeable 3.7-V battery pack (Meircell, Ashdod, Israel) of dimensions 67.1 × 55.2 × 18.5 mm, weight 145 g and capacity 7800 mAh, sufficient to power the recording device for approximately eight days of uninterrupted recording. The battery pack was fitted with a 2-pin Molex-type female connector in order to connect to the recording device. The device operated under RockBox software 1.2.7 (https://www.rockbox.org/, accessed on 5 January 2025) which added the critical functionality of recording to the MicroSD memory card, and the automatic, gapless saving of files of specified size or duration, in a choice of formats. The device was configured to store recordings on a 32-GB MicroSD card as WAV-format files of 90 min duration, at a sampling frequency of 48 kHz. In field tests, the inward-facing microphone recorded sound vibrations generated by jaw movements and transmitted via the skull to the horn of the cow, with virtually no surrounding noise.

For protection, the recording device was housed in a customized aluminum casing (A. Braun Metals, Tel-Aviv, Israel) of dimensions 61.6 × 41.4 × 15.6 mm and weight 73.6 g, with an aperture for the two pairs of cables. This was stacked on the external battery and placed inside a larger customized casing of dimensions 74.2 × 61.1 × 35.2 mm and weight 179.5 g, with an aperture for the microphone cable. Following assembly and the initialization of recording, the casing was waterproofed with heavy-duty duct tape. The total weight was 425 g.

### 2.6. Acoustic Sensor Deployment

Prior to installation on an animal, and after removal, a set of taps was recorded indicating the precise date–time, allowing later correction for clock drift. At installation, each animal in the group was restrained in a cattle squeeze, with its head immobilized for operator safety. The box-shaped protective casing containing the recording device and battery was taped around one horn approximately 4 cm from its base and securely fastened to the horn using a turn-key metal hose clamp (Ideal Tridon, Smyrna, TN, USA; diameter 152.4 mm). The external microphone was then taped to the horn in the space between the casing and its base, with the vibration pad facing inward. The entire assembly was wrapped well with heavy-duty duct tape (Supplementary Figure S8). At the end of the monitoring period (after approximately one week), the animals were similarly restrained for the removal of the equipment.

It was imperative that the sensor should not detectably alter the animal’s behavior or impinge on its welfare by virtue of its size or weight. Three measures were taken to verify this. First, on release from the squeeze, the animals were retained in a small holding area for approximately 30 min in order to observe them closely for any indication that the apparatus was bothering the animals (e.g., head-shaking, pacing, general irritation). Second, all animals were inspected and observed during active grazing every day of the monitoring periods. Third, a series of 21 video recordings of 5 min duration were made of a random selection of the experimental cows during active grazing to verify that behavior was normative.

### 2.7. Processing of Acoustic Data

The large database of audio files was analyzed automatically by an algorithm that was developed in house and described in Navon et al. [29]. The algorithm reliably identifies sound-generating jaw movements but does not classify them. Algorithmically, detecting jaw movements is easier and more precise than classifying them [23,30,31,32]. The algorithm was designed to be as general as possible in terms of animal species, foraging environment and recording equipment, and to require no calibration. The algorithm identifies jaw movements according to key features in the time domain that are defined in relative terms and uses a machine-learning approach to separate true jaw movement (JM) sounds from spurious background noise. One feature of true JMs is that the signal intensity undergoes a sharp change at the start and end of the sound burst. Second, true JMs rarely occur in isolation as “singles” but occur as part of a sequence. Third, the sound signal intensity of bites and chews is strong compared to any background ambient noise that might be detected. Fourth, in cattle, there is a limited range of duration within which all normative sound-generating jaw movements fall. Combining these criteria enables low rates of false positive and false negative events to be achieved, especially when working with vibration-type microphones, as in the present study. Preprocessing of the signal includes correction for the clock drift of the recording device, high-pass filtering (60 Hz), amplification and down-sampling to reduce computational load. The algorithm outputs the timeline of unclassified JM events. To monitor the performance of the algorithm, the waveform of the sound signal was overlaid with the timeline of JM events identified by the algorithm, and at least 30 validation segments per season–animal, of 10 min duration each, were examined closely for false negative and false positive events.

### 2.8. The Core Datasets

The raw dataset for the spring season (March) deployment contained ≈1.6 M JM events obtained from 11 of the 12 recording devices that each yielded ≈100 h of continuous recording from installation. (The twelfth device failed to record soon after deployment.) The recording duration was shorter than expected due to poor battery performance. The signal quality was consistently poor in one device, and its data were excluded. The stream of JM events from the remaining 10 devices/cows was trimmed: data from the day of installation (early morning until midnight) were excluded, as were all data after midnight 72 h later. The remaining three complete 24 h cycles, starting from midnight, were retained for analysis. The dataset contained 1,203,568 JM events, including rumination.

The raw dataset for the summer season (July) deployment contained ≈3.9 M JM events obtained from 11 recording devices that each yielded ≈180 h of continuous recording. (One device failed early, but battery performance was in the expected range for the remainder.) By way of trimming, data from the early morning until 14:00 h on the day of installation were removed, as were all data after 14:00 h, seven complete 24 h cycles later. In one device, the data for the last 24 h cycle were incomplete; thus, the data for that entire day were excluded. The resultant dataset, drawn from 11 cows, contained 3,652,708 JM events, including rumination.

### 2.9. Designation of Rumination Bouts

The most important distinction that needed to be made in the JM timelines was that between rumination JMs and the remainder, with all the latter being regarded as ingestive JMs. This can be performed manually by scanning the timeline of JM events for the distinctive pattern generated by rumination: a long, repetitive sequence of highly regular events (within-bolus chews) interspersed at regular intervals by short, few-second (inter-bolus) gaps. However, when using screens of a fixed time span, the number of screens to scan is high and many of them contain few, if any, events. It was found to be more efficient *and informative* to scan interval-based plots, whereby JM events are arranged sequentially along the *x*-axis, and the time interval between successive events (previous to current) is shown on the *y*-axis. For each season–device combination, the intervals were computed from the second JM event until the last. For visual-scanning purposes only, the data were arbitrarily divided into 1000-event groupings that were displayed graphically in a series of plot panels, each showing 1000 values across the full width of the screen, with sufficient resolution to identify individual events. The time interval (*y*-axis) was displayed on a logarithmic scale.

For the most part, the visual identification of rumination bouts was rapid and unequivocal. It is inconceivable that such distinctively regular rhythms of sound bursts and interruptions could be generated by chance during grazing; thus, aural verification that all the JMs were exclusively pure chews was not required. Small deviations or transient fluctuations in the pattern, or occasional longer breaks in the order of 1 min, were tolerated as part of a single rumination bout. The interval associated with the first chew action of a bout was designated as part of grazing and not rumination. Using features of the graphic interface of JMP v16 (SAS Institute Inc., Cary, NC, USA), all such clearly identifiable bouts of rumination (comprising multiple boluses) were designated “rumination” in a dedicated pass of the 1000-event plot panels. Individual boluses were not delineated. Runs of JM events to be designated as rumination could begin or end at any position in a plot panel and could span multiple panels.

There were patterns that retained features of rumination but deviated significantly from the idealized level of regularity, perhaps caused by transient periods of poor signal quality that degraded event identification. There were also rumination-like patterns that were not sustained sufficiently to make the designation definitive, as they may have occurred by chance during grazing. Both these types of pattern were designated “possible rumination” in a separate, dedicated pass of the 1000-event plot panels. All jaw activity not defined as rumination or possible rumination was assumed to be associated with ingestion.

### 2.10. Designation of Additional Patterns

In the process of scanning the interval-based plots to designate rumination, it became clear immediately that “grazing”, i.e., anything not designated as rumination or possible rumination, does not have a universal “signature” pattern as does rumination. Although the same baseline rhythm of interval values of ≈1 s dominated grazing, the interruptions inserted into it were highly erratic. Nevertheless, three fairly distinct patterns were recognizable among the non-rumination sections, representing opposite extremes of JM intensity and something in the middle. Since these patterns emerged organically from the data and were not a planned part of the methodology, they are described in detail in the Results (Section 3). Bouts of the two extreme patterns were marked up in separate, dedicated passes of the 1000-event plot panels. All remaining events, representing the majority of non-rumination JM events, then received a single designation. Note that certain patterns included long intervals (in the order of 10^3^ s), which would conventionally be called “rest”. Rather than drawing an arbitrary line between “rest” and “graze” intervals, we treated the entire (non-ruminating) day as a set of intervals that can be considered collectively as “grazing” in a broader sense of the term, even though they span orders of magnitude.

### 2.11. Rate-Based Analysis

The timeline of JM data was summarized as minutely counts (within season, device and day number). This generated a file of 109,761 records, which was then zero-padded to account for “quiet” minutes and ensure a record for every minute in the time range of the data for each season and device (*n* = 152,640 records). Non-zero counts were subdivided according to the five-way designation of patterns (two for rumination and three for grazing), and a minutely designation was assigned based on the maximum. Quiet (zero-padded) minutes were designated as such. The resulting file was used for all rate-based analyses and to generate empirical cumulative distribution function (CDF) curves.

## 3. Results

### 3.1. Sensor Impact on Animal Welfare

All 12 experimental animals appeared to tolerate the horn-based deployment of the acoustic sensor very well. Based on close observation immediately after installation, the animals appeared indifferent to the sensor within a couple of minutes of release from the cattle squeeze. No aberrant behavior was observed in the holding area. The daily observations of grazing never detected a deviation in head orientation or pattern of movement from what is normative. Likewise, careful review of the video recordings of grazing found no change from normative grazing behavior. None of the devices became dislodged during deployment or required an intervention.

### 3.2. Signal Quality and Algorithm Performance

**Figure 1:**
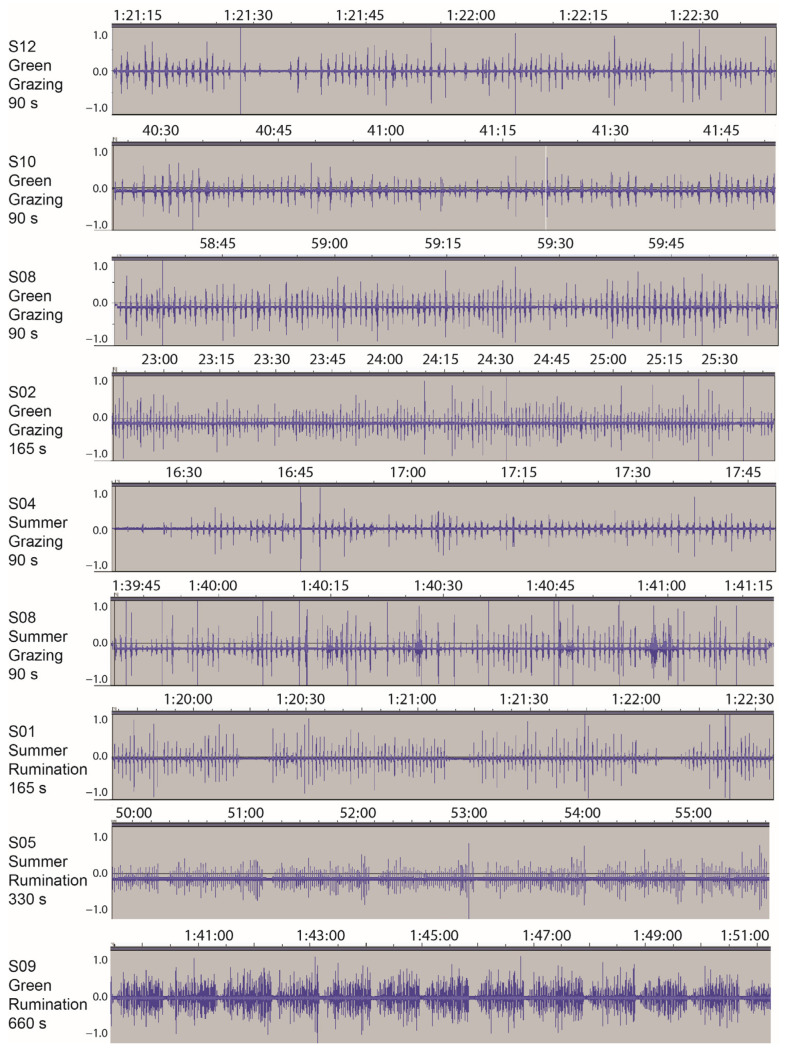
Examples of the waveform obtained via the acoustic monitoring of grazing beef cattle cows on a Mediterranean herbaceous rangeland in 2013. The waveforms represent the pattern of sound pressure variation (amplitude) in the time domain. Where apparent, *y*-axis offsets were caused by amplification. The panel labels show the cow designation, the season of recording, activity (grazing or rumination) and the duration of the sample shown. The green and summer season samples were recorded on 22 March and 13 July, respectively. Times are hh:mm:ss or mm:ss.

For the overwhelming majority of time recorded, the quality of the signal was high, and the occurrence of jaw movements that performed a bite or chew action were readily identifiable aurally. The chew–bite type of jaw movement, with its characteristic rapid-fire pair of sound bursts, was ubiquitous. Examples of waveforms obtained from different animals are shown in Figure 1, mostly at a scale at which individual jaw movement events can be resolved. The algorithm performed well in identifying sound-producing jaw movements, and transient noises were discriminated well. Based on all the validation segments examined, the false positive and false negative rates were 3% and 4%, respectively, which are within the expected limits of the algorithm [29]. Despite the fact that dry vegetation tends to elicit a weaker acoustic signal than green vegetation, false positive and false negative error rates were similar across seasons.

### 3.3. Overview of the Data

The complete database of JM events contained 4,856,276 records that were sourced from a total of 106 cow–days over two seasons. In the broadest terms, and as a rough frame of reference, the ratio of total JMs to cow–days was ≈46,000 daily events, which could be subdivided fairly equally into ≈22,000 (48%) rumination events (including possible rumination) and ≈24,000 (52%) ingestion-related (i.e., all other) events. Assuming a normative, unimpeded rate of jaw movement (RJM) during both active grazing and rumination to be ≈60 min^−1^, in the best-case scenario of machine-like jaw activity at the normative rate, the animal would spend ≈6 h d^−1^ ruminating and ≈7 h d^−1^ grazing. The global sum of the intervals associated with rumination was equivalent to ≈7 h cow–day^−1^, quite close to the “ideal” value. Deriving a comparable estimate for grazing time is problematic because “grazing” as used here really means non-rumination, and includes pauses, interruptions and breaks of *any* duration.

**Figure 2:**
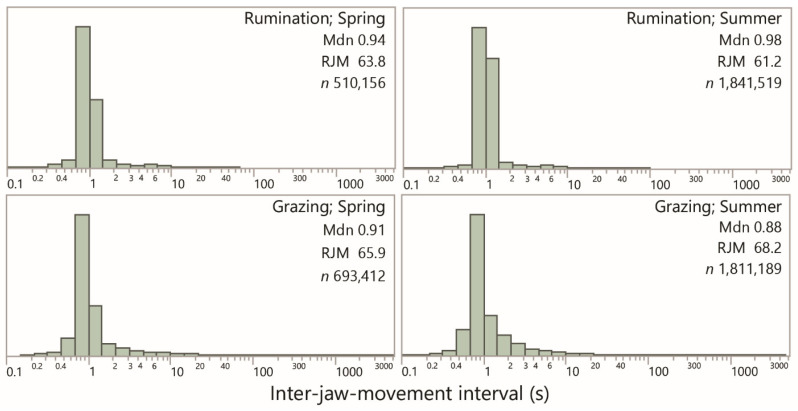
Frequency distributions of the inter-jaw movement interval (s) for rumination and grazing (i.e., non-rumination) in the spring and summer seasons. Values are the median of the distribution (Mdn), the equivalent rate of jaw movement (RJM; min^−1^) and the number of intervals in the sample (*n*). Uniform logarithmic scaling has been applied across panels. The lowest end of the distribution is predominantly associated with false positive identifications by the algorithm.

A total of 4876 interval-based plots were visually scanned. Almost all of them were dominated overwhelmingly by a band of interval values of ≈1 s. Figure 2 shows the frequency distribution of the inter-event interval for rumination and grazing, according to season, using logarithmic scaling for interval. All the distributions share a similar dominant (“modal”) interval, approximated (and slightly overestimated) by the median, which ranged from 0.88 s to 0.98 s among the four combinations shown. The variability of intervals was much lower for rumination (SD ≈ 1 s) than for grazing (SD ≈ 35 s), consistent with the fact that this perspective on jaw activity bypasses distinctions between grazing and resting in the conventional sense.

### 3.4. Rhythms, Patterns and Styles of Jaw Activity

#### 3.4.1. Rumination

**Figure 3:**
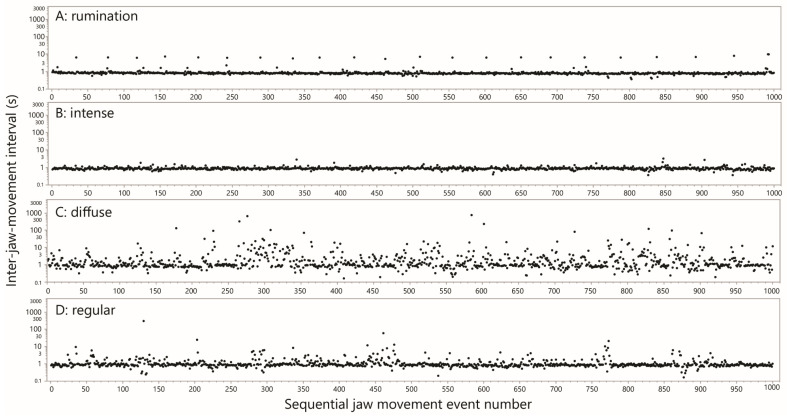
Representative examples of interval-based plots of jaw movement activity for the different designations. Jaw movement events are arranged sequentially along the *x*-axis, and the time interval between successive events is given on the *y*-axis (log scale). For display purposes, the events were arbitrarily divided into groupings of 1000. Uniform logarithmic scaling has been applied across all panels. Panel (**A**) includes within it the rumination waveform for cow S09 in Figure 1, and Panel (**B**) includes within it the green season grazing waveform for cow S08 in Figure 1.

For the most part, the interval-based plots facilitated recognition of rumination bouts with a high degree of certainty, primarily by virtue of the consistent rhythm and magnitude of the inter-bolus break. Rumination bouts presented as a recognizable, distinctive pattern of runs of very similar values of ≈1 s, jumping periodically to a single interval of ≈8 s, as shown in Figure 3A. In total there were 2,250,193 JM events designated as rumination, and a further 101,482 events designated as possible rumination. After merging these into a single designation, the total number of rumination bouts was 1470, or 13.9 bouts cow–day^−1^ (in general, a bout is defined as an uninterrupted run of JMs of the same designation, bounded at each end by a JM of a different designation). The mean number of chew events per bout was 1600, and the mean duration of a bout was 1803 s (30 min).

**Figure 4:**
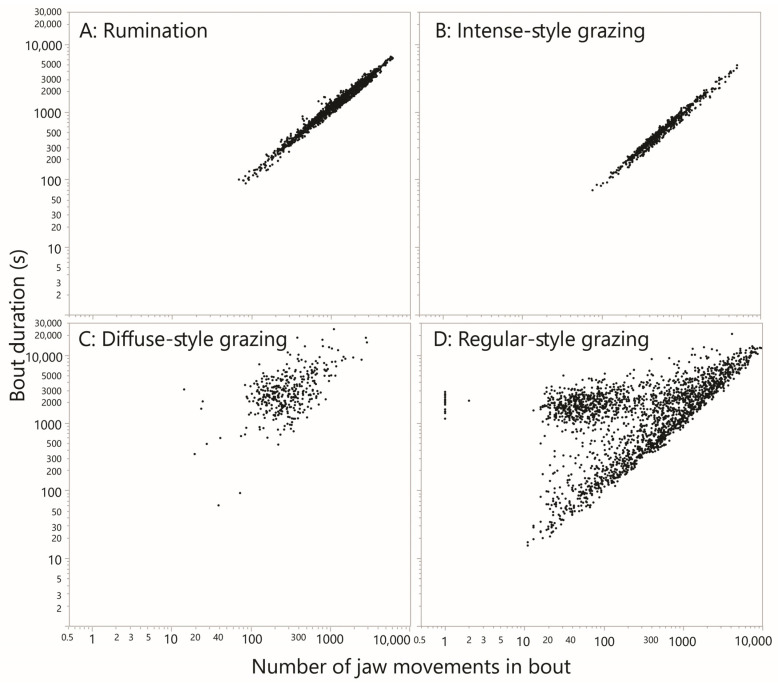
The relationship between the duration of a bout of jaw activity (*y*-axis; s) and the number of jaw movements performed (*x*-axis; dimensionless) for rumination bouts and for bouts of the three grazing styles that were designated. A bout is defined as an uninterrupted run of jaw movements of the same designation. EquaI log–log scaling has been applied in all panels.

The linear regression between the bout duration and bout event count explained 97% of the variation; the slope was 1.05 s event^−1^, equivalent to 57 JMs min^−1^ (Figure 4A). Because the bout duration included many inter-bolus intervals, the slope value slightly underestimated the natural (within-bolus) JM frequency of rumination. In order to examine whether rumination bouts tended to be preceded by long breaks, the sum of the inter-JM interval for the previous 20 events to the designated start of a rumination bout was computed. Likewise, to examine whether rumination bouts tended to be succeeded by long breaks, the sum was computed for 20 events starting at the end of a rumination bout. This allowed for imprecision in the demarcation of the transition to and from rumination. The frequency distributions of the preceding and succeeding breaks were broadly similar, scaling logarithmically and reaching ≈4000 s (Supplementary Figure S9). Both distributions had a strong modal region, centered at ≈40 s and ≈25 s for the preceding and succeeding breaks, respectively. Those modal values are sums over 20 events and therefore imply almost gapless transitions.

#### 3.4.2. Intense Grazing

This pattern stood out in the scans as an uninterrupted run of hundreds of JMs for which almost all intervals were ≈1 s, with infrequent “interruptions” to this rhythm by longer intervals of up to a few seconds. These runs of JMs, which typically contained ≈1000 events and frequently traversed plot panels, were designated “intense”. Much shorter runs (<200 events) would only be marked if they exhibited unusually low variation. At the other extreme, slightly longer “interruptions” were tolerated if they enabled significantly longer runs to be marked. A total of 731 intense-style runs of JMs were designated, comprising 548,047 JM events. An example of an intense-style run is shown in Figure 3B. The time duration of intense-style runs and the number of JM events contained therein were highly correlated, as would be expected (Figure 4B). With all the data included in the analysis, the slope of the relationship was 0.92 s event^−1^, equivalent to 65 JMs min^−1^. The median interval of the pooled intense runs was 0.84 s, equivalent to 71 JMs min^−1^. These runs can be interpreted only as representing intense grazing. Notably, the total time devoted to intense-style grazing was ≈139.6 h, or 1.3 h cow–day^−1^, accounting for only 23% of the daily ingestive JMs performed. The remainder must have required many more additional hours of less intense jaw activity, lending credence to the notion of behavioral grazing intensity.

#### 3.4.3. Diffuse Grazing

The grazing pattern designated “diffuse” presented as a relatively diffuse and disorderly mix of intervals in the range up to ≈10 s, dotted with a number of much larger intervals that could reach 10^2^ and 10^3^ s (Figure 3C). A total of 378 diffuse runs of JMs were designated, comprising 134,080 JM events. The time duration of such a run and the number of JM events contained therein were correlated much less strongly (Figure 4C) than they were for intense-style grazing. The median time duration of a diffuse run was 2724 s (45 min), and the median number of JM events contained therein was 262. Nevertheless, the median interval of the pooled diffuse runs was 1.2 s, not much greater than that of intense-style runs. However, whereas for intense-style runs ≈80% of values were <1 s, for diffuse-style runs it was only ≈40%. Diffuse-style runs are interpreted as short bursts of uninterrupted JMs, interspersed with interruptions of highly variable length that reach ≈1 h. The total time devoted to diffuse-style grazing was ≈373 h, or 3.5 h cow–day^−1^.

#### 3.4.4. Regular

All events not designated as rumination, possible rumination, intense-style grazing or diffuse-style grazing were designated as regular. The interval-based pattern of regular-style runs of JMs resembled the intense style but were interrupted irregularly by eruptions of longer intervals. An example of a regular-style run is shown in Figure 3D. A total of 2158 regular-style runs were designated, comprising 1,822,474 JM events. The time duration of such a run and the number of JM events contained therein were weakly correlated (Figure 4D). There also appear to be two populations that create a forked pattern, and this will be addressed in the Discussion (Section 4.5). The median interval of the pooled regular runs was 0.90 s, or 67 JMs min^−1^. The total time devoted to regular-style grazing was ≈1294 h, or 12.2 h cow–day^−1^.

### 3.5. The Rate-Based Perspective

**Figure 5:**
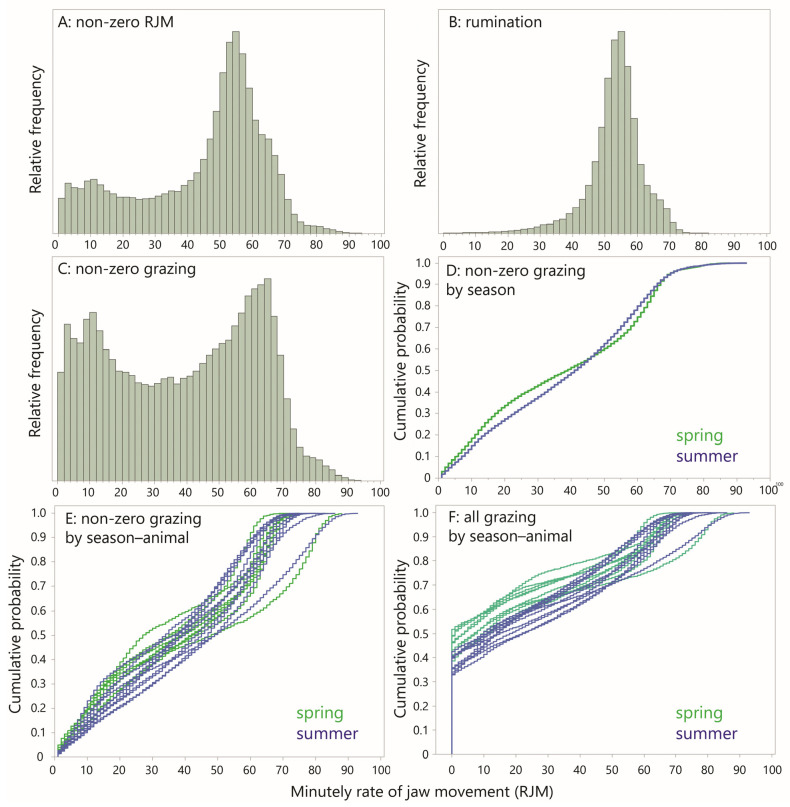
Frequency distributions (FDs) and empirical cumulative distribution functions (CDFs) for minutely rate of jaw movement (RJM). (**A**) FD of all non-zero RJM values; (**B**) FD of rumination only; (**C**) FD of all non-zero “grazing” (non-rumination); (**D**) CDF of all non-zero “grazing” RJM by season; (**E**) CDF of all non-zero “grazing” RJM, for each season–animal combination; (**F**) CDF of all “grazing” RJM, including zero values, for each season–animal combination.

The frequency distribution of the pooled dataset of all RJM values was dominated by zero-RJM values, and these will be considered separately. For all non-zero RJM values, the frequency distribution of the pooled dataset containing both rumination and grazing showed a strong central tendency in the region of the median (51 JM min^−1^) but was not symmetrical; below the median, there was a sizeable representation over the entire range, with an indication of a secondary mode in the low RJM range (Figure 5A). The frequency distribution of RJM values for minutes designated as rumination showed a more symmetrical shape, with a similar median (53 JM min^−1^) and no elevated presence in the low range (Figure 5B).

The non-zero, grazing RJM values that remained after the exclusion of all the rumination designations (there were no 1 min level designations of possible rumination) are clearly the source of the bimodality in the RJM (Figure 5C). Nevertheless, the dominant mode of ≈62 JM min^−1^ is greater than that of rumination (≈55 JM min^−1^) and is 90% of the “ideal conditions” comparator cited in the Introduction (Section 1). Given that ≈20% of RJM values shown exceeded 62 JM min^−1^, it is clear that the animals were able to sustain episodes of intense grazing that operated at a near-potential RJM, despite the relatively poor forage conditions of Mediterranean rangeland. But the sizable proportion (approximately half) of RJM values in the range below 40 JM min^−1^ lends further credence to the notion of behavioral grazing intensity. This is only reinforced by the strong bimodality of the distribution, having a secondary peak in the region of 10–20 JM min^−1^, suggestive of a second, “light grazing” mode. There was some difference between the seasons in the degree of bimodality, as can be seen by comparing their CDF curves (Figure 5D). This seasonal contrast was also apparent in the CDF curves of the individual animals, but there was some overlap (Figure 5E).

The seasonal difference in the CDF curve becomes more strongly apparent if the zero-RJM values (“quiet” minutes) are included (Figure 5F). The zero-RJM values form the initial vertically ascending section of the curves, showing a clear seasonal contrast, with more “quiet” minutes in the spring. The number of zero-RJM values per 24 h period was 499 (8.3 h) in the spring and 367 (6.1 h) in the summer, a difference of 2.2 h.

### 3.6. Diurnality

**Figure 6:**
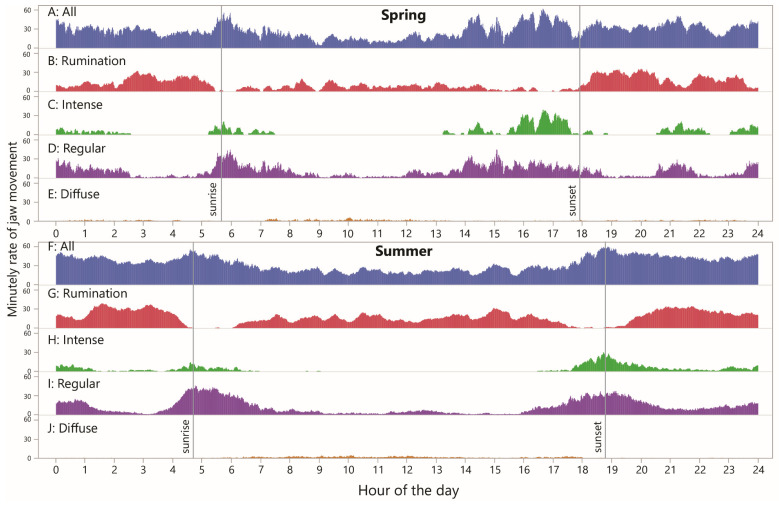
Minutely rate of jaw movement (RJM) over the course of 24 h, in total and by designation, averaged over all cow–days within season. Uniform scaling has been applied throughout.

**Figure 7:**
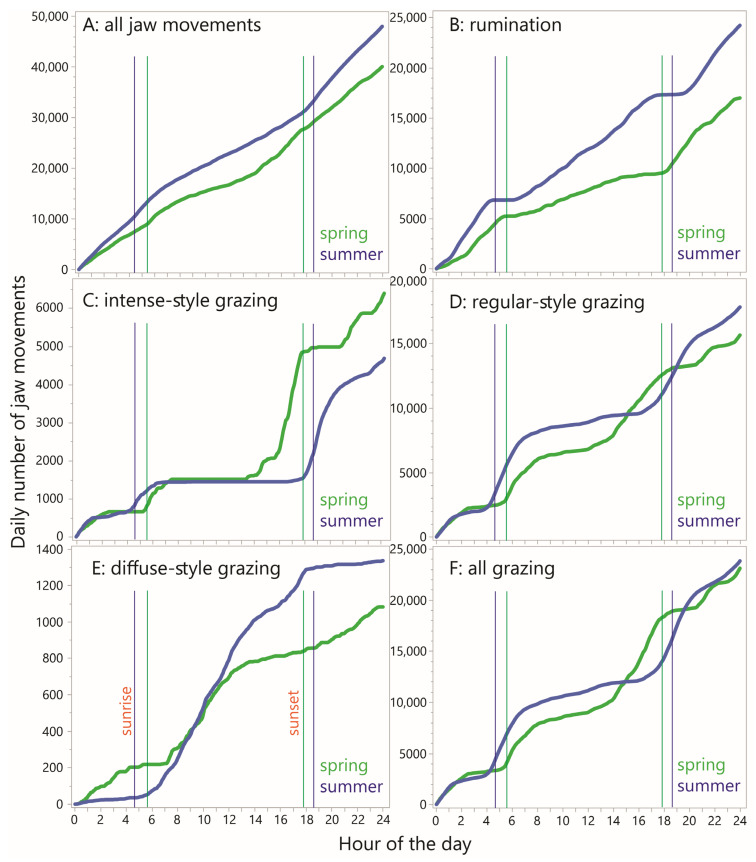
Cumulative distribution functions (CDFs) of minutely rate of jaw movement (RJM) over the course of 24 h, averaged over all cow–days within each season, according to designation. *Y*-axes are scaled independently.

To examine diurnality in jaw activity, the RJM values (including zeros) were averaged by minute-in-day across all the animal–days within the season (30 and 76 animal-days in spring and summer, respectively). This was performed for total JM, rumination JM and the three grazing styles, as shown in Figure 6. Generally, the values appeared higher in the summer than in the spring (Figure 6A,F), confirmed by the CDF curves of daily total JMs (Figure 7A). The pattern for total JM showed a modest overall reduction in the RJM in the late morning and early afternoon hours in the spring, and a similar pattern over a wider window in the summer.

The corresponding pattern for rumination was somewhat similar, showing more activity at night than in the central part of the daylight hours, at least in the spring season, but there were narrow windows of time with a very low likelihood of rumination occurring (Figure 6B,G). These time windows appear as relatively flat sections on the CDF curves, which also show that a large part of the difference in total JMs stems from a difference in total rumination JMs, being considerably higher in the summer (Figure 7B).

The diurnal pattern for “intense” jaw activity showed a high degree of localization, with zero or close-to-zero activity over a period of six (spring) or nine (summer) consecutive hours of the daytime, and somewhat separate windows of activity during the remainder (Figure 6C,H). In the spring, there was a long nighttime window, from ≈2300 h to ≈0230 h, in which at least *some* animals were engaged in intense-style grazing in every minute (pooling sampling days). The fact that this pattern in particular was found to occur at night highlights the importance of night grazing. The most prominent window of intense-style grazing occurred between ≈1530 h and 1730 h, with average the RJM reaching a maximum of 40 min^−1^. In the summer, the afternoon waves of activity seen in the spring were absent, and the main activity wave of the day occurred between ≈1730 h and 2100 h. These trends are well reflected in the shape of the CDF curves (Figure 7C), which also show a large gap in the peak value reached, to the advantage of the spring season.

As seen for intense-style grazing, the diurnal pattern for regular-style grazing was also organized into windows of elevated activity, and these overlapped with, and were wider than, the more disconnected windows of intense-style grazing (Figure 6D,I). There was some level of regular-style grazing throughout the 24 h period and its diurnal pattern was broadly complementary to that of rumination. The CDF curves show a similar nighttime wave of activity, followed by a much larger daybreak wave in the summer than in the spring, and a similar but shifted (delayed) wave towards evening (Figure 7D). The total regular-style JMs was somewhat greater in the summer season.

Regarding diffuse-style grazing, it becomes clear when viewed in a rate-based way that this designation contributes extremely little to total JMs and is more aptly described as intervals interrupted sporadically by jaw activity, rather than the reverse (Figure 6E,J). The steepest sections of the CDF curves started with daybreak but extended much later into the day in the summer and were relatively absent in the night in the summer, in contrast to the spring season (Figure 7E).

## 4. Discussion

### 4.1. Beyond the Purely Technical

It is some 35 years since Alkon et al. [33], working with porcupines, drew attention to the potential of acoustic monitoring in the study of animal behavior. Since then, various groups have independently applied the concept to domesticated livestock (e.g., [23,32,34]), often with rudimentary equipment. They have demonstrated proof-of-principle in terms of the physical device and its installation on the animal, and in terms of automatic signal processing. We are not aware of a commercial acoustic sensor system for the monitoring of jaw activity, and such monitoring has not become a routine component of grazing research or management; it is actually rare. Size matters in the collection of such data if it is to function as a benchmark description of grazing behavior for a particular environment, and to the best of our knowledge, this is the largest database of jaw movement activity published to date (see also Vanrell et al. [35] and Martinez-Rau et al. [36]). But the motivation for further development of the technology is strengthened if the mass of data generated can be used to provide a penetrating and sensitive profile of how the animal interacts with its forage environment.

### 4.2. A Rest-Less View of Jaw Activity

The interval-based perspective of jaw activity presented here bypasses conventional distinctions between grazing and rest. That is not to deny that animals rest. The intention was to avoid the imposition of arbitrary thresholds on the data and to examine whether some pattern emerges organically. Non-ruminatory jaw activity is conceptualized as comprising an underlying rhythm of the natural jaw movement frequency (≈60 min^−1^) that is fractured by interruptions, the duration of which scales logarithmically. Thus, behavioral grazing intensity is determined by the mix of interruptions that are inserted into the jaw movement stream. Both the diffuse-style and regular-style patterns included large intervals that would conventionally be defined as rest, but here such intervals were treated as an integral part of the jaw movement stream. In fact, dropping the graze–rest distinction yields a more holistic expression of what the animal does.

Our estimate of the natural baseline frequency was 71 min^−1^ for intense-style grazing, remarkably similar to the mean RJM of 69 min^−1^ cited in the Introduction (Section 1), based on Ungar and Rutter [20], to represent ideal grazing conditions. The values obtained in the present study sit well within the cloud of points assembled from various sources [8,12,20,28,35,37,38,39] that shows the natural RJM in relation to live weight (Supplementary Figure S10). Note that in the present study and in the study by Tani et al. [28], the natural RJM is lower in rumination than in grazing/ingestion. We ascribe this to ruminatory mastication having a greater rotational motion in the jaw movement cycle [40], which has a time cost.

### 4.3. The Non-Binarity of Grazing

It can be argued that traditional measurements of grazing time are a reasonable surrogate for intake if grazing is binary in nature (yes or no), *and* jaw movement rate when “yes” is fairly constant. The perspective that emerged here did not support the binary view. Nevertheless, that does not preclude setting thresholds for transition from grazing to rest and vice versa, recognizing that they are somewhat arbitrary (this is explored below in Section 4.6). But the second condition of the RJM being constant was certainly not met: non-zero minutely RJM values were spread over the entire feasible range and even displayed bimodality. The fact that we distinguished between intense and regular styles of grazing does not prove that such distinct styles exist. In contrast, the clear bimodality of the frequency distribution of a non-zero RJM *does* suggest that grazing is not a simple (possibly normally distributed) continuum of jaw movement intensity. In this sense, grazing cannot be viewed as binary, and a highly variable RJM rate degrades the utility of grazing-time estimates. It also complicates our ability to obtain unbiased estimates in field observations.

### 4.4. Seasonal Differences

**Figure 8:**
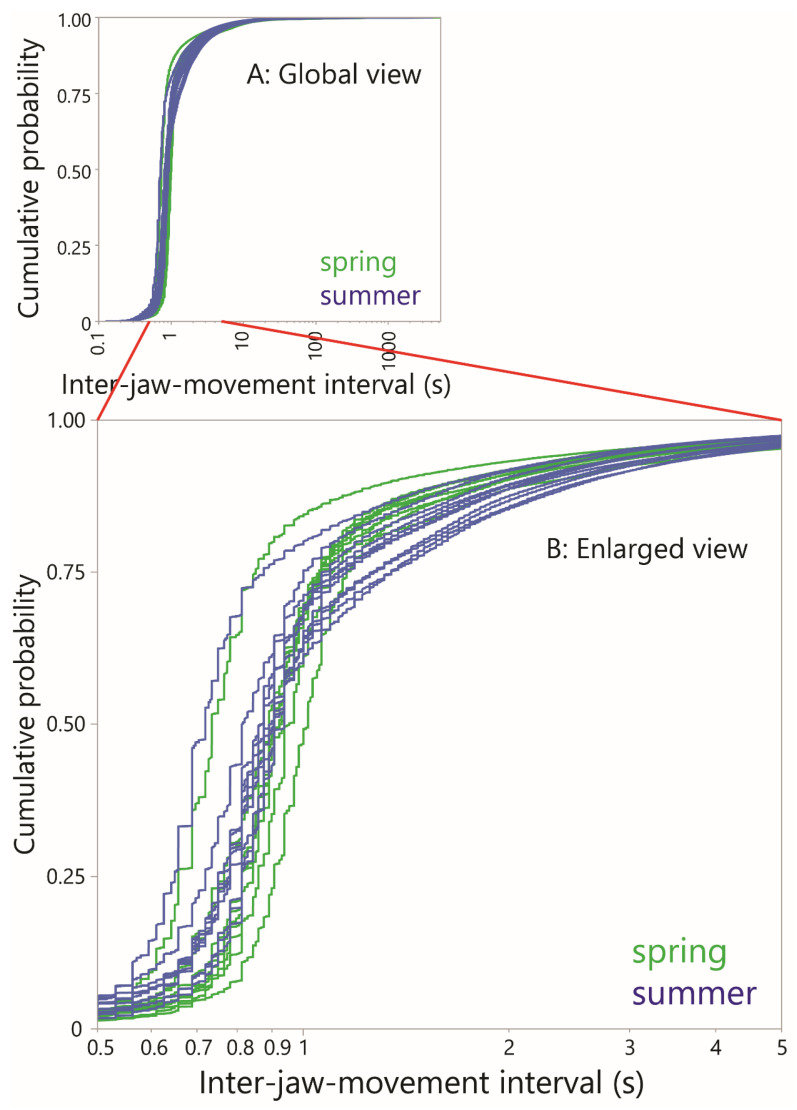
Cumulative distribution functions (CDFs) of inter-jaw movement interval for all grazing (i.e., non-rumination), plotted at the animal level within each season. (**A**) Global view; (**B**) Enlargement in the region 0.5 s to 5 s.

The sensitivity of the interval-based approach can be seen in the empirical CDF curves of the inter-JM interval. These were constructed to compare the fine structure of jaw activity between seasons (Figure 8). This showed that below an interval of 1 s, which is greater than the natural jaw movement frequency, the two groups of curves were completely intermingled. But over the range 1–3 s they mostly (but not exclusively) separated out: the spring season curves rose more steeply and plateaued earlier than the summer curves, which increased more gradually over this 2 s range. The curves of the two seasons reconverged at higher intervals. One possible explanation is that in the summer, subtle changes occur in the intake process involving a greater search component at the fine spatial scale and, more speculatively, slower bite formation in order to select bites that avoid stemmy material.

### 4.5. Substructure

A feature of our data that requires explanation is the forked pattern obtained in Figure 4D for regular-style grazing. The relatively horizontal cloud of points that is somewhat separated from the main relationship shows a fairly constant total duration in the region of 2000 s, being achieved by a number of jaw movements centered near 50, i.e., extremely few JMs min^−1^. The main trend in Figure 4D shows that ≈2000 JMs would be performed in such a duration. These interludes suggest the existence of a natural “relative rest” period that lasts approximately half an hour, containing relatively high interval values. We are confident these interludes were not an artifact produced by the carving out of intense-style grazing runs. If that were the case, the forked pattern would disappear if intense-style and regular-style grazing runs were assigned a common designation. This was not observed; the forked pattern persisted after the re-designation (Supplementary Figure S11). Note that although high interval values are uncommon, they are potent in terms of their effect on the timeline of jaw activity. To account for this, the following final step in the analysis portrays the *entirety* of the population of intervals shown in Figure 8 in a modified way.

### 4.6. The Time Accumulation Curve

**Figure 9:**
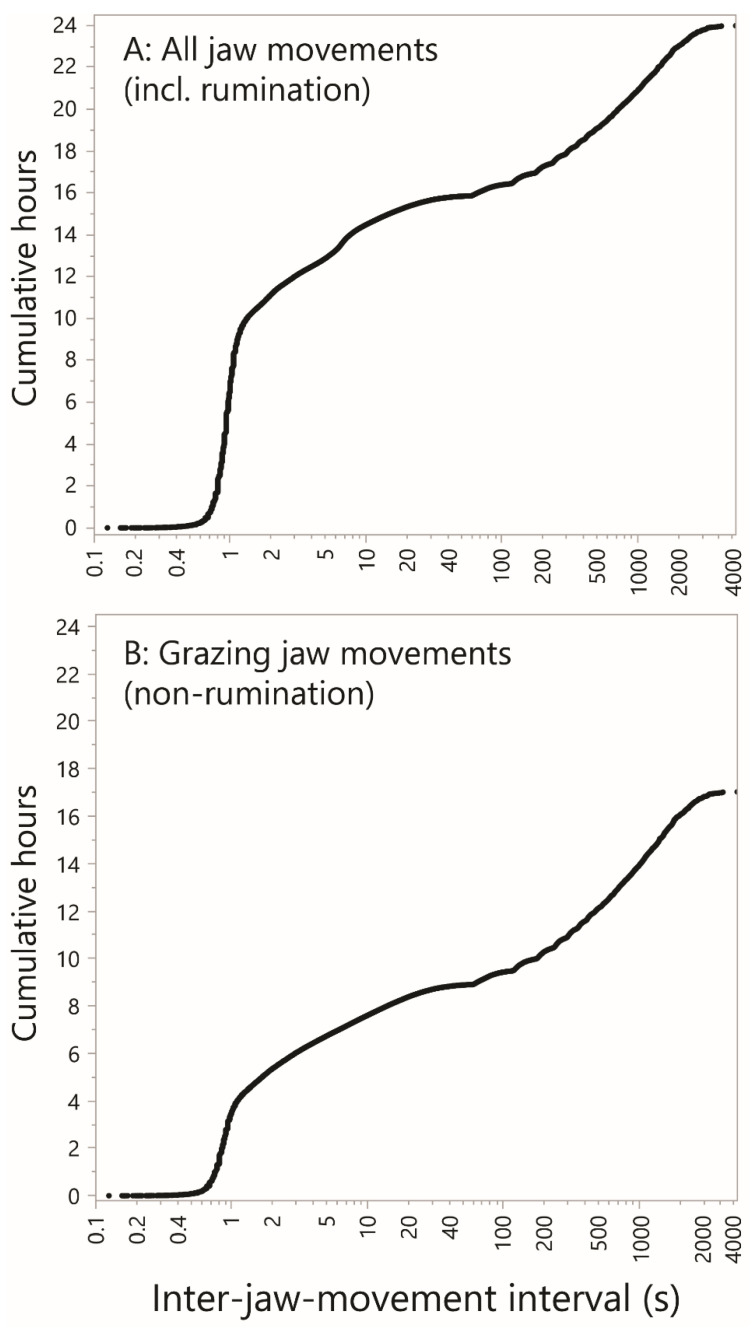
The cumulative time spent performing jaw movements in relation to inter-jaw movement interval. The *y*-axis is the cumulative, ascending ranked, inter-jaw movement interval, expressed in hours per cow–day. (**A**) All jaw movements, including rumination. (**B**) Grazing (i.e., non-rumination) jaw movements. Uniform scaling has been applied throughout.

A CDF-like curve was constructed that shows how the inter-jaw movement intervals contribute to total time accounted for, essentially weighting each inter-JM interval by its own size. The value of interest is the cumulative, ascending-ranked, inter-jaw movement interval, expressed in h cow–day^−1^. This is shown as a function of the inter-jaw movement interval, on a logarithmic scale, to yield what we term the empirical time accumulation curve. When *all* jaw movements were included in the analysis (Figure 9A), intervals up to a value slightly greater than 1 s account for 9 h of daily activity. The curve then continues to rise less steeply, and fairly smoothly, on the logarithmic scale. There is a minor inflection in the region of 7 s, and it is fair to assume that the local steep rise in the curve is caused by the highly consistent inter-bolus interval of rumination. But there is a major inflection in the region of 40–100 s as the curve transitions from a relatively flat zone in the range 30–60 s to a relatively steep zone in the region of 1000–2000 s, consistent with the “relative rest” suggested above. Based on this, it could be argued that there is a natural separation of inter-jaw movement intervals into those associated with grazing and those associated with rest, with the transition in this inflection region. If a 1 min cut-off is used, grazing and rumination together occupied ≈16 h of the day.

When rumination is excluded, the curve shown in Figure 9B is obtained. Here, only 4 h of daily activity is accounted for by intervals up to a value slightly greater than 1 s. The minor inflection point associated with the inter-bolus interval is absent. The general shape of the remainder of the curve remains unchanged since it contains virtually no rumination-related intervals. Using a 1 min cut-off, grazing occupied ≈9 h of the day, and the animal would be deemed to be at rest for the remaining 8 h of the day. The question raised here is how the shape of the entirety of the curve shown in Figure 9 responds to different foraging environments. That will require the integration of large jaw movement datasets from multiple sources.

## 5. Conclusions

Much can be learned from just the timeline of unclassified jaw movement events, especially when acoustic monitoring is conducted continuously over multiple days. The main insights gained can be summarized as follows.

1. Jaw activity reveals rhythms, patterns and styles. Rumination bouts were unique in combining strong *rhythm*—shown by the within-bolus runs of chew events all falling within a narrow interval band of ≈1 s—with a strong *pattern* created by the regular insertion of fairly constant inter-bolus intervals, many multiples of the baseline interval, at a larger time scale.

2. There was no single “signature” jaw movement pattern for grazing (i.e., all non-rumination JM events). Although a similar underlying “natural” rhythm seen during rumination dominated the non-rumination population of intervals, it was intermittently and irregularly interrupted by longer intervals *whose size scaled logarithmically.*

3. There was evidence of further substructure showing a degree of separation between “grazing” and “resting” in the conventional sense of the terms. This revealed itself as two zones of relatively steep ascent in the time accumulation curve: one in the region of the natural, unimpeded RJM, with an interval of ≈10^0^ s, and one in the region of 10^3^ s, which would appear to correspond to rest in the conventional sense.

4. The distribution of non-zero ingestive JM rates shows strong bimodality. The primary modal region was centered near the frequency corresponding to the natural rhythm of ≈60 JM min^−1^, demonstrating that jaw movement rate on natural rangelands can match that achieved on abundant sown pastures. The secondary modal region was centered at ≈15 JM min^−1^, suggesting a different mode of grazing. Beyond speculation, it is unclear how to explain the bimodal nature of grazing intensity, and what benefit low-intensity grazing brings.

5. The notion of behavioral grazing intensity is supported strongly by the rate-based results. This calls into question the approach of viewing grazing as a binary state or expecting measures of grazing time to be indicative of intake rate.

Rate- and interval-based analyses of information at the jaw movement level have furnished a useful way of profiling how an animal interacts with its foraging environment, culminating in the time accumulation curve. The classification, and not just identification, of jaw movements would be expected to yield greater insights, which is a major reason why acoustic monitoring holds much promise. These findings would seem to justify further development of the acoustic monitoring technology to enable its wider adoption in grazing research and management.

## Data Availability

Data will be made available on request.

## Supplementary Materials

The following supporting information can be downloaded at: https://www.mdpi.com/article/10.3390/s25041210/s1, Figure S1: Images from the observational study, three from the spring season and three from the summer season; Figure S2: Daily rainfall in the study region for hydrological year 2013/2014; Figure S3: Time course of 10-min-interval temperature in the study region covering the periods of acoustic monitoring in the spring and summer seasons; Figure S4: Time course of 10-min-interval global radiation in the study region covering the periods of acoustic monitoring in the spring and summer seasons; Figure S5: Time course of 10-min-interval relative humidity in the study region covering the periods of acoustic monitoring in the spring and summer seasons; Figure S6: Time course of 10-min-interval wind direction in the study region covering the periods of acoustic monitoring in the spring and summer seasons; Figure S7: Time course of 10-min-interval wind speed in the study region covering the periods of acoustic monitoring in the spring and summer seasons; Figure S8: An experimental cow moments after release from the cattle squeeze following installation of the acoustic sensor on one horn; Figure S9: Frequency distribution of pre- and post-rumination zone events, based on the sum of the 20 events preceding or succeeding the labelled start or end of a rumination bout; Figure S10: The relationship between rate of jaw movements (RJM) and live-weight for cattle grazing naturally or otherwise; Figure S11: The relationship between the duration of a bout of jaw activity and the number of jaw movements performed for grazing bouts (pooled across grazing styles).

## Abbreviations

CDF = cumulative distribution function; FD = frequency distribution; JM = jaw movement, RJM = rate of jaw movement.
