# Supplementary material for: Rhythms, Patterns and Styles in the Jaw Movement Activity of Beef Cattle on Rangeland as Revealed by Acoustic Monitoring
Source: Sensors (Basel). 2025 Feb 17;25(4):1210. doi: 10.3390/s25041210 (PMC11860019; doi:10.3390/s25041210)
Supplement: Supplementary file 1 [file sensors-25-01210-s001.zip › sensors-3442944-supplementary.pdf]

Supplementary Figures S1 -- S11

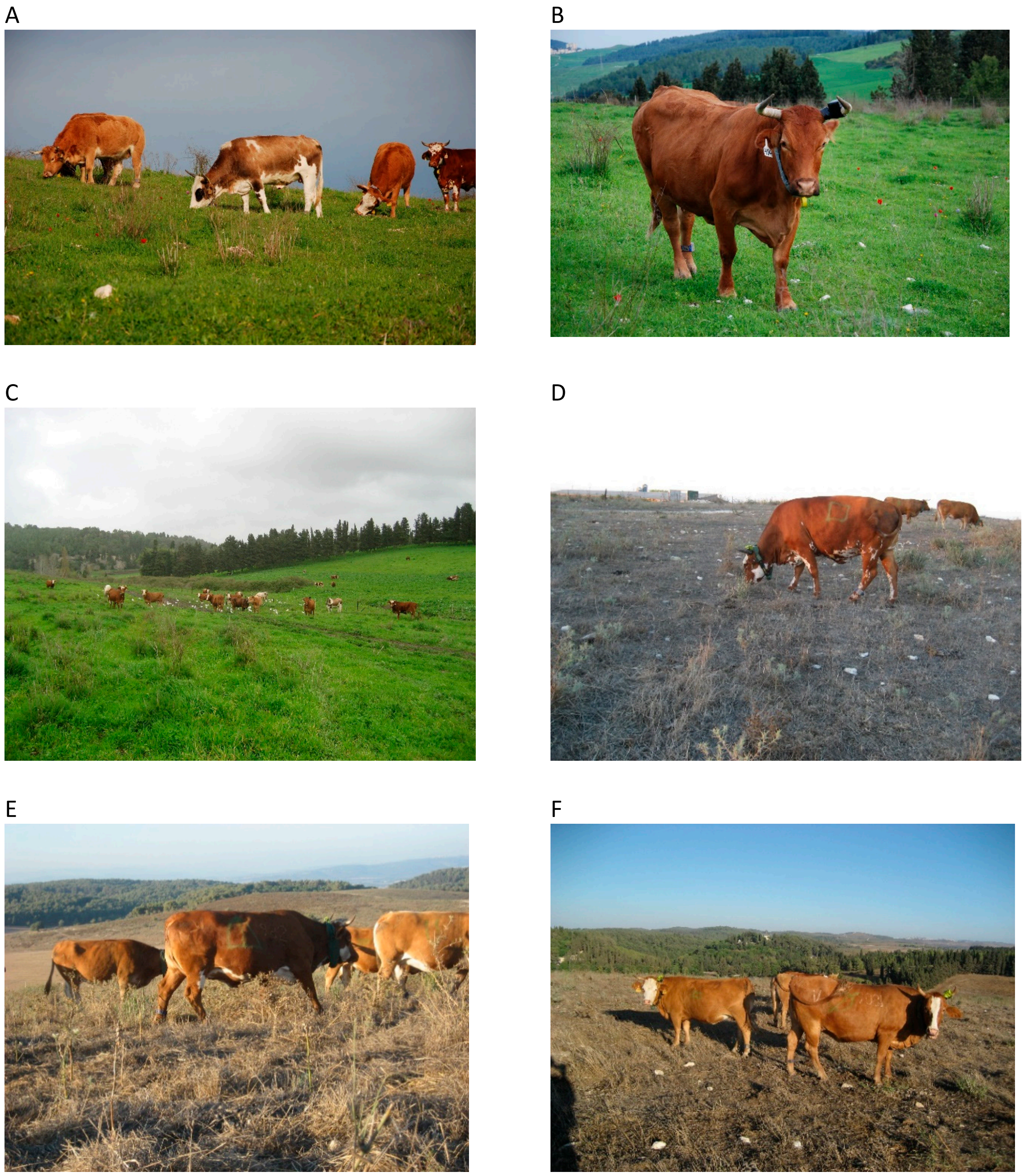

Figure S1. Images from the observational study, three from the spring season (panels A, B, C) and three from the summer season (panels D, E, F). The horn-mounted acoustic monitoring equipment can be seen clearly in panel B.

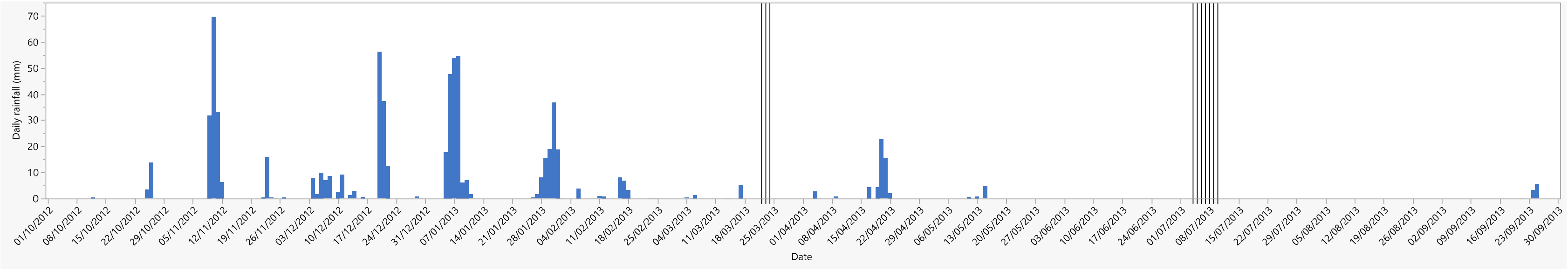

Figure S2. Daily rainfall in the study region (Ein Hashofet meteorological station) for hydrological year 2013/2014. Vertical lines show the dates of acoustic monitoring in the spring and summer seasons used in the analysis.

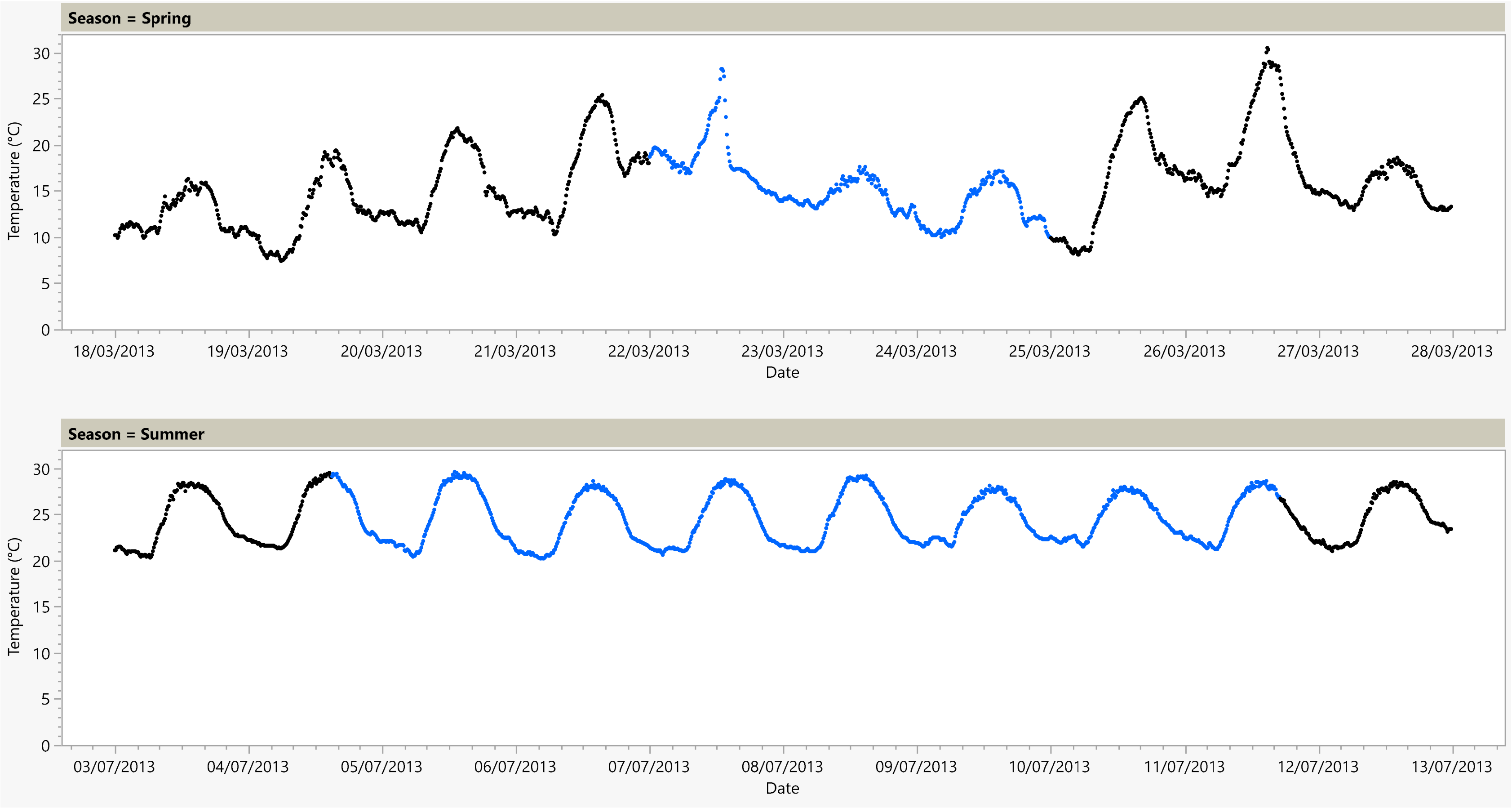

Figure S3. Time course of 10-min-interval temperature in the study region (Ein Hashofet meteorological station) covering the periods of acoustic monitoring in the spring (upper) and summer (lower) seasons. In the spring there were three 24-h cycles of continuous monitoring used in the analysis, and in the summer, there were seven such cycles (shown in blue). Mean daily temperature in the spring and summer monitoring periods was 15.3 °C and 24.5 °C, respectively.

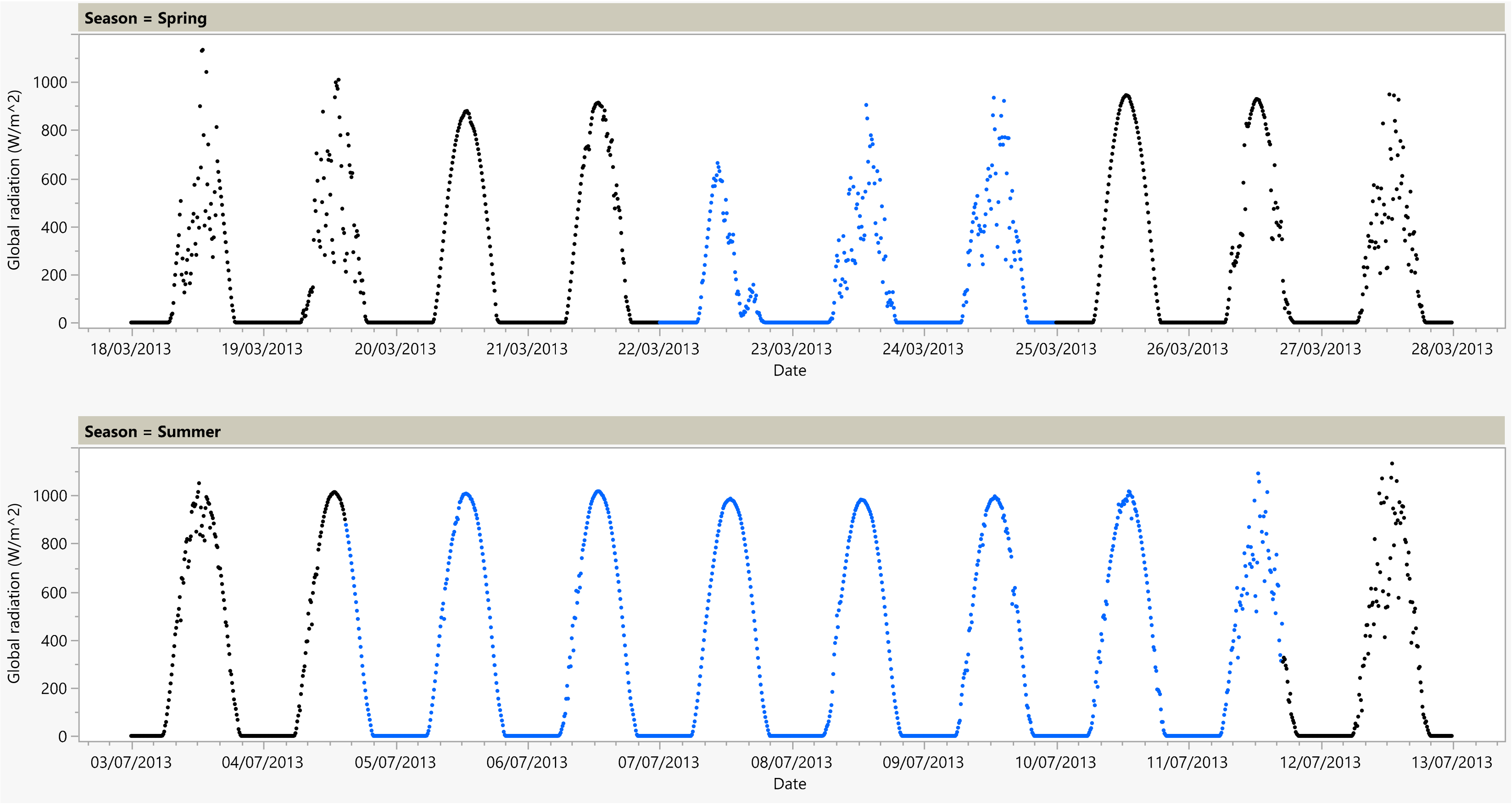

Figure S4. Time course of 10-min-interval global radiation in the study region (Ein Hashofet meteorological station) covering the periods of acoustic monitoring in the spring (upper) and summer (lower) seasons. In the spring there were three 24-h cycles of continuous monitoring used in the analysis, and in the summer, there were seven such cycles (shown in blue). Mean global radiation in the spring and summer monitoring periods was 168.5 W m^{-2} and 339.3 W m^{-2}, respectively.

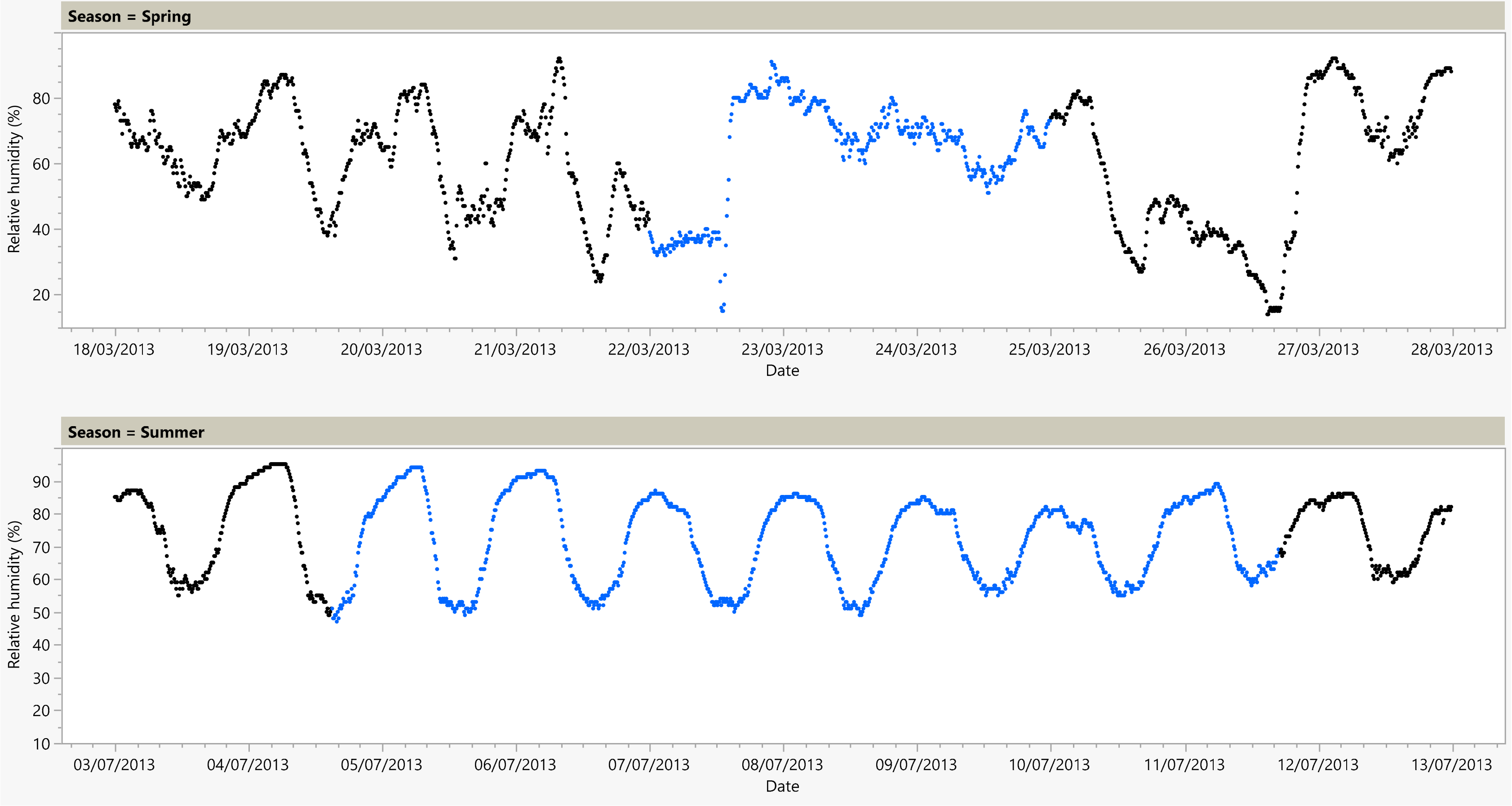

Figure S5. Time course of 10-min-interval relative humidity in the study region (Ein Hashofet meteorological station) covering the periods of acoustic monitoring in the spring (upper) and summer (lower) seasons. In the spring there were three 24-h cycles of continuous monitoring used in the analysis, and in the summer, there were seven such cycles (shown in blue). Mean relative humidity in the spring and summer monitoring periods was 64.0% and 71.5%, respectively.

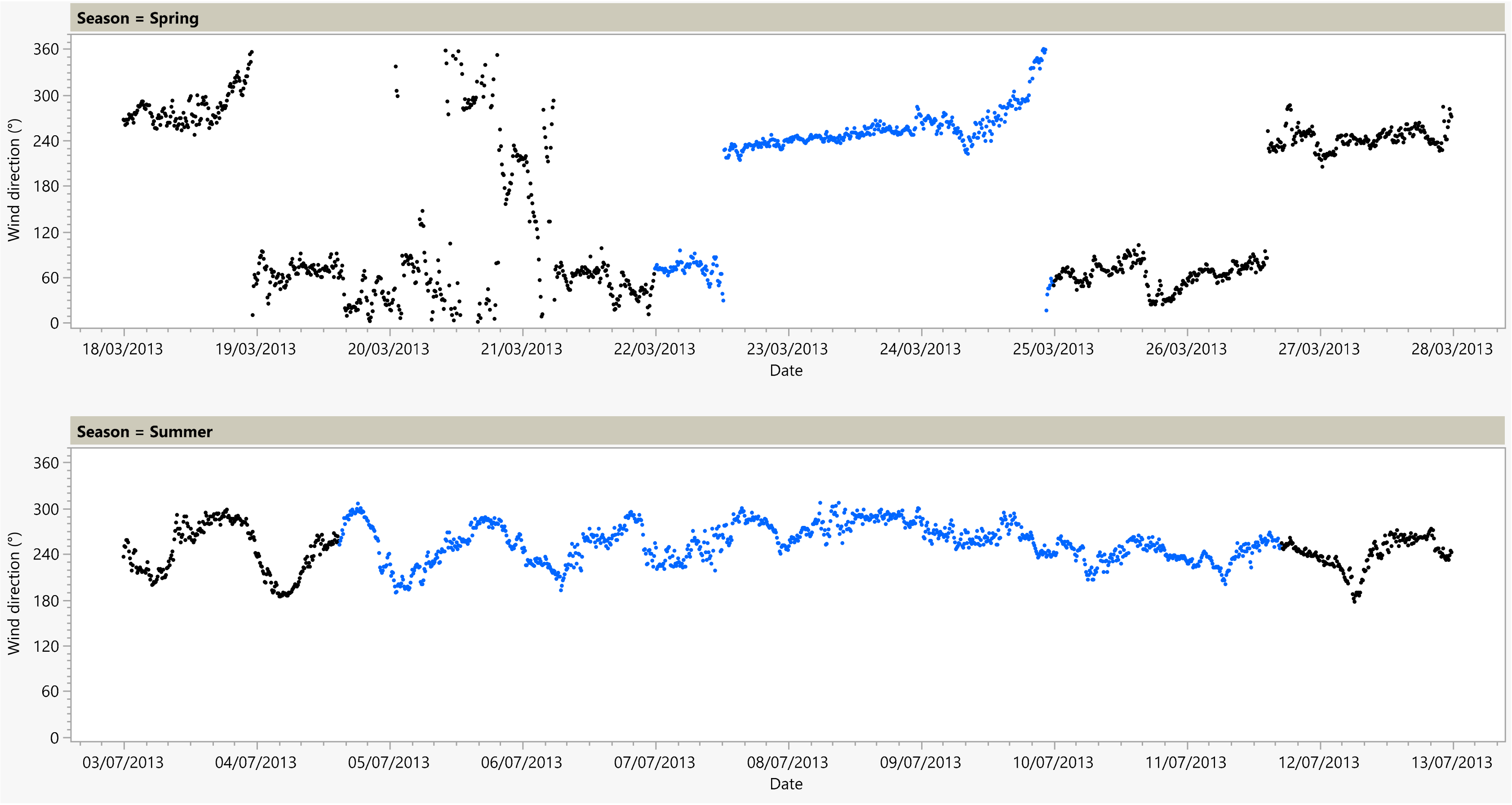

Figure S6. Time course of 10-min-interval wind direction in the study region (Ein Hashofet meteorological station) covering the periods of acoustic monitoring in the spring (upper) and summer (lower) seasons. In the spring there were three 24-h cycles of continuous monitoring used in the analysis, and in the summer, there were seven such cycles (shown in blue).

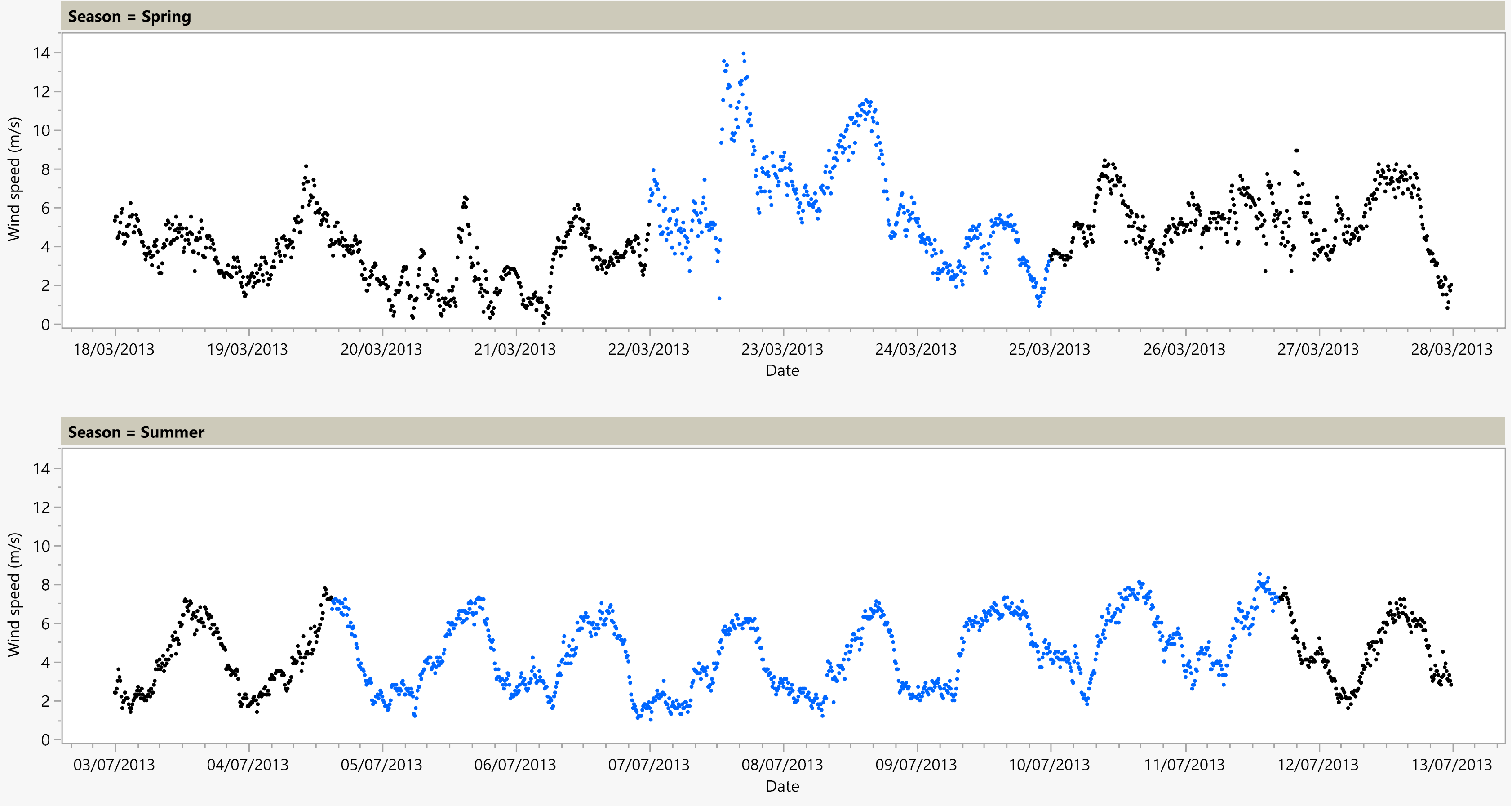

Figure S7. Time course of 10-min-interval wind speed in the study region (Ein Hashofet meteorological station) covering the periods of acoustic monitoring in the spring (upper) and summer (lower) seasons. In the spring there were three 24-h cycles of continuous monitoring used in the analysis, and in the summer, there were seven such cycles (shown in blue). Mean wind speed in the spring and summer monitoring periods was 6.2 m s^{-1} and 4.4 m s^{-1}, respectively.

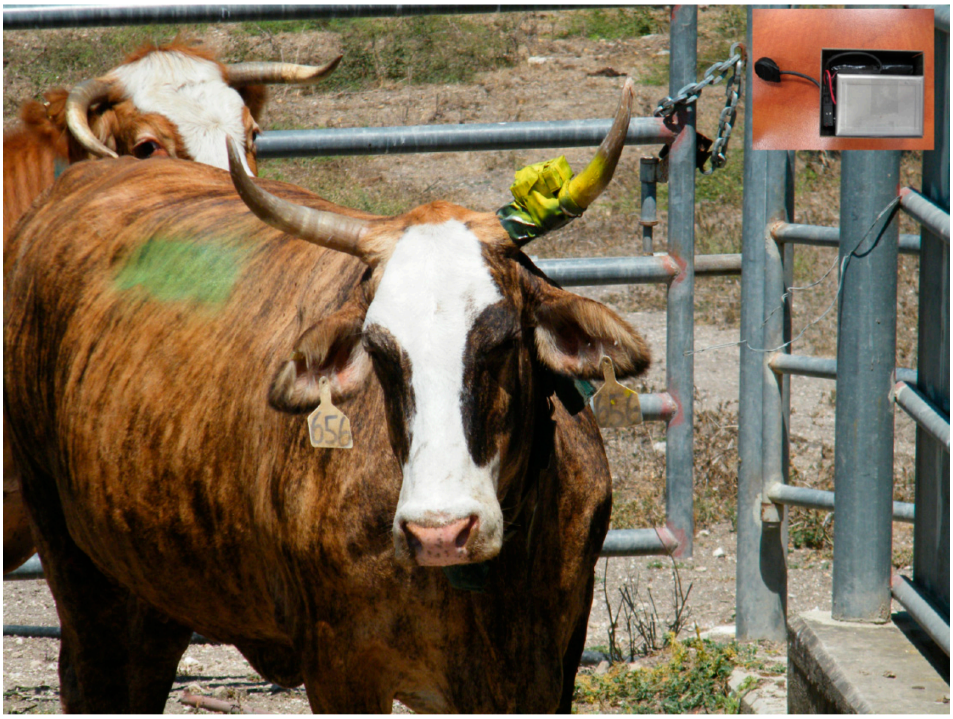

Figure S8. An experimental cow moments after release from the cattle squeeze following installation of the acoustic sensor on one horn. Yellow spray paint was used to ease identification in the field. The inset shows the equipment prior to closure of the outer protective box.

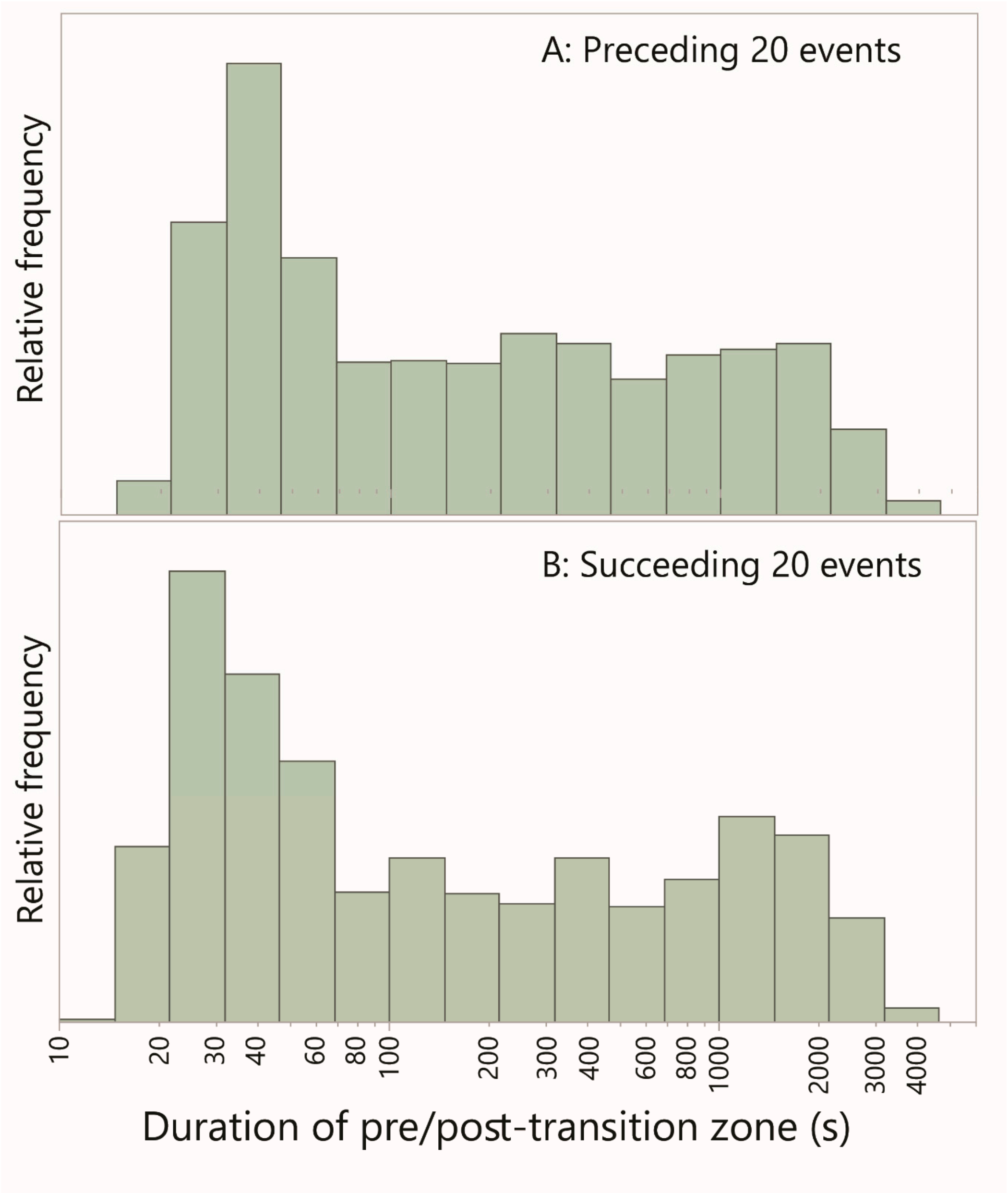

Figure S9. Frequency distribution of pre- and post-rumination zone events, based on the sum of the 20 events preceding or succeeding the labelled start or end of a rumination bout. A: Pre-rumination break; B: Post-rumination break.

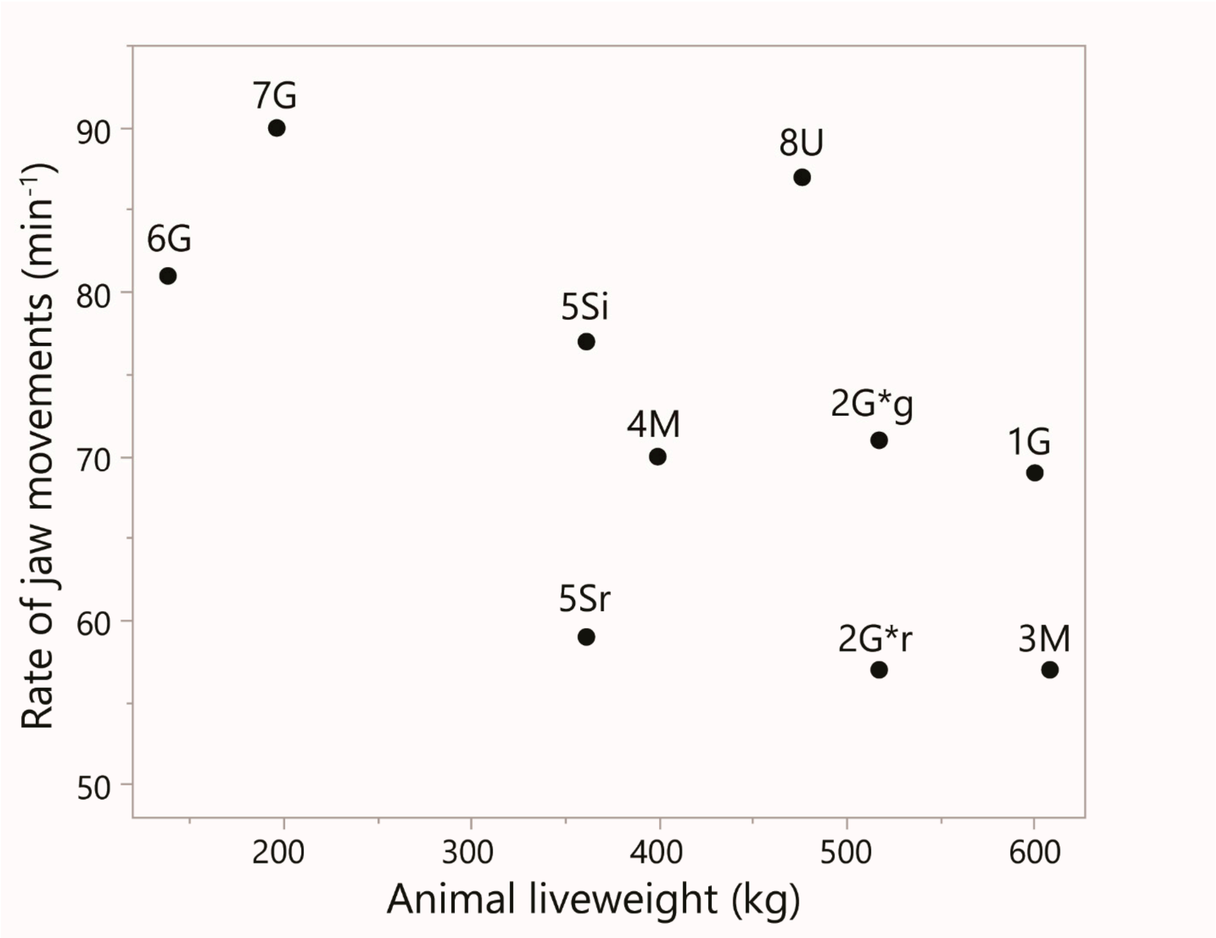

Figure S10. The relationship between rate of jaw movements (RJM) and live-weight for cattle grazing naturally or otherwise. 1G = Ungar and Rutter [20]; 2G*g = present study, grazing; 2G*r = present study, rumination; 3M = Galli et al. [38] and Vanrell et al. [35] for dairy cows grazing microswards; 4M = Yayota et al. [12] for cows grazing microswards; 5Si = Tani et al. [28] for silage consumption; 5Sr = Tani et al. [28] for rumination; 6G = Nadin et al. [37] for grazing calves; 7G = Nadin et al. [39] for grazing steers; 8U = Druzinsky [8], zoo feeding of *Bos taurus*.

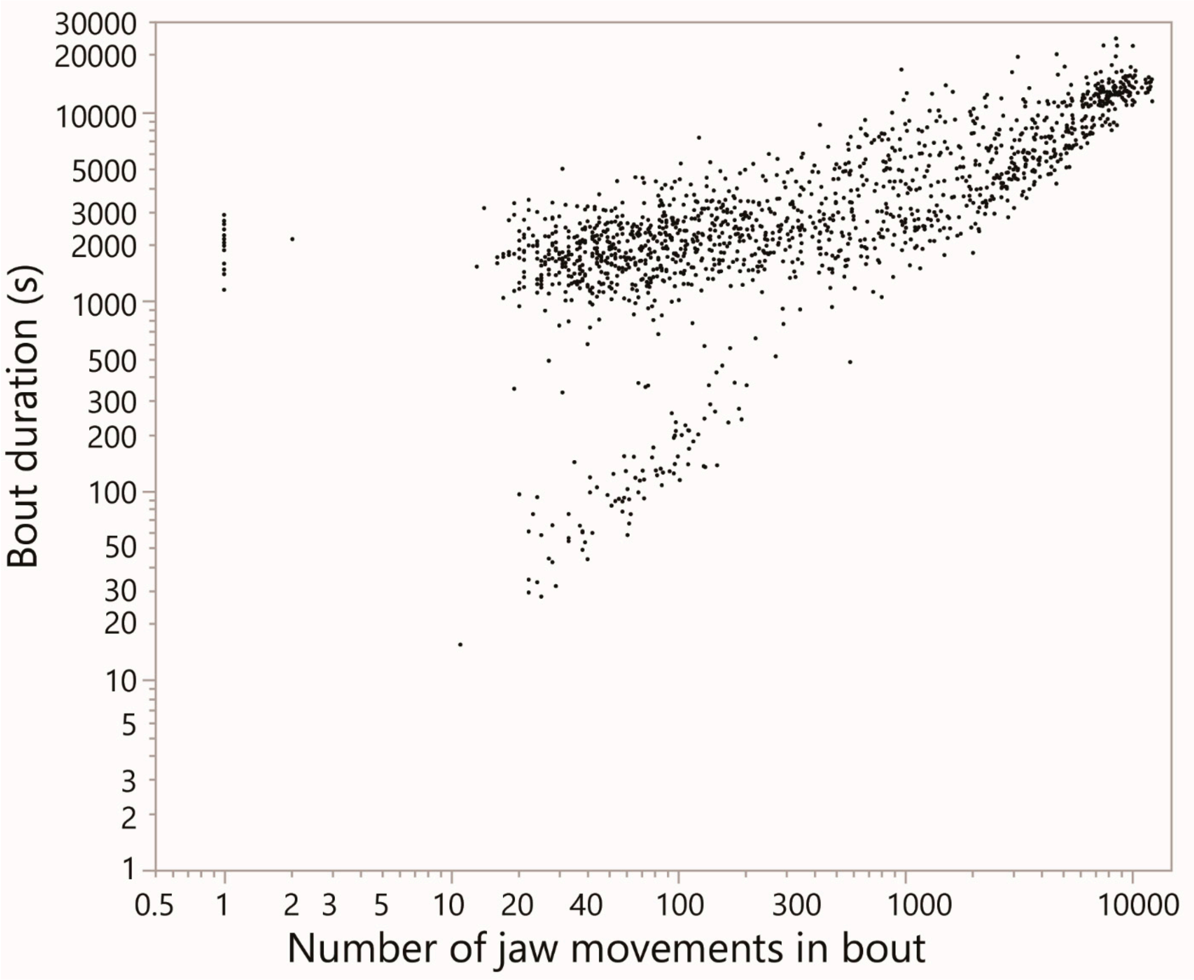

Figure S11. The relationship between the duration of a bout of jaw activity (*y*-axis; s) and the number of jaw movements performed (*x*-axis; dimensionless) for grazing (i.e., non-rumination) bouts (pooled across grazing styles). Log-log scaling has been applied as in Figure 4.
